# Frontier knowledge and future directions of programmed cell death in clear cell renal cell carcinoma

**DOI:** 10.1038/s41420-024-01880-0

**Published:** 2024-03-05

**Authors:** Cao Fei, Xu Zhen, Zhang Shiqiang, Pang Jun

**Affiliations:** 1 Department of Urology, Pelvic Floor Disorders Center, The Seventh Affiliated Hospital, Sun Yat-sen University, Shenzhen, Guangdong 518107 China; 2 Scientific Research Center, The Seventh Affiliated Hospital, Sun Yat-sen University, Shenzhen, Guangdong 518107 China

**Keywords:** Renal cell carcinoma, Renal cell carcinoma

## Abstract

Clear cell renal cell carcinoma (ccRCC) is one of the most common renal malignancies of the urinary system. Patient outcomes are relatively poor due to the lack of early diagnostic markers and resistance to existing treatment options. Programmed cell death, also known as apoptosis, is a highly regulated and orchestrated form of cell death that occurs ubiquitously throughout various physiological processes. It plays a crucial role in maintaining homeostasis and the balance of cellular activities. The combination of immune checkpoint inhibitors plus targeted therapies is the first-line therapy to advanced RCC. Immune checkpoint inhibitors(ICIs) targeted CTLA-4 and PD-1 have been demonstrated to prompt tumor cell death by immunogenic cell death. Literatures on the rationale of VEGFR inhibitors and mTOR inhibitors to suppress RCC also implicate autophagic, apoptosis and ferroptosis. Accordingly, investigations of cell death modes have important implications for the improvement of existing treatment modalities and the proposal of new therapies for RCC. At present, the novel modes of cell death in renal cancer include ferroptosis, immunogenic cell death, apoptosis, pyroptosis, necroptosis, parthanatos, netotic cell death, cuproptosis, lysosomal-dependent cell death, autophagy-dependent cell death and mpt-driven necrosis, all of which belong to programmed cell death. In this review, we briefly describe the classification of cell death, and discuss the interactions and development between ccRCC and these novel forms of cell death, with a focus on ferroptosis, immunogenic cell death, and apoptosis, in an effort to present the theoretical underpinnings and research possibilities for the diagnosis and targeted treatment of ccRCC.

## Facts

- A new regulatory form of cell death, cuproptosis, has recently been reported in the journal Science, further reinforcing the importance of cell death in living organisms.
- With the deepening of the understanding of programmed cell death, more and more studies have shown that different programmed cell death (such as ferroptosis, immunogenic cell death, apoptosis, etc.) are closely related to the occurrence and development of kidney cancer.
- Inducing ferroptosis will significantly inhibit the invasion and metastasis of kidney cancer, and is closely related to the better prognosis of kidney cancer patients.
- Apoptosis can not only induce the death of kidney cancer cells, but also activate the immune response against kidney cancer.
- Immune-targeted therapy based on immunogenic cell death is the main treatment modality for advanced kidney cancer.

## Questions

- What are the connections of each mode of cell death to ccRCC?
- How to improve or propose anti-cancer approaches based on programmed cell death?

## Introduction

Renal cell carcinoma (RCC) is the third most prevalent malignancy in the genitourinary system following prostate and bladder cancer [1]. Clear cell renal cell carcinoma (ccRCC) is responsible for an estimated 70–85% of RCC with higher invasive ability and relapse than other RCC subtypes [2]. Upon initial diagnosis, approximately one-third of RCC patients present with metastatic lesions. Furthermore, following radical surgical intervention, an estimated 30% of RCC cases initially categorized as non-metastatic undergo the development of distant metastases [3, 4]. Treatment strategies for advanced ccRCC include novel kinase inhibitors, and a combination of checkpoint inhibitors plus targeted therapies [5]. However, drug resistance remains a primary challenge in treating metastatic ccRCC, and the current elucidation of drug-resistant mechanisms is insufficient to overcome clinical resistance [6].

The emergence of resistance to cell death mechanisms is a common phenomenon observed in drug-resistant tumor cells [6]. Enhanced comprehension of the responsible mechanisms of cell death might result in the discovery of new potential targetable pathways [7]. Accidental cell death is the uncontrolled process of cell death that is usually triggered by unexpected stimuli that exceed the cell’s ability, which includes regulating its metabolic processes, repairing damaged components, and appropriately respond to external signals to avoid excessive or uncontrolled cell death, to regulate. While programmed cell death (PCD) is an active process associated with unique biochemical characteristics, morphological features, and immunological profiles [8]. The combination of immune checkpoint inhibitors (ICI) plus targeted therapies is the first-line therapy recommended by National Comprehensive Cancer Network and European Society of Medical Oncology [9]. Nivolumab and ipilimumab, two ICIs that target CTLA-4 and PD-1, induce T cell activation and cause tumor cells death by binding to B7 ligands CD80 and CD86 expressed on antigen-presenting cells (APCs) and PD-L1 on either tumor cells or APCs, respectively [10]. Axitinib, a VEGFR inhibitor, leads to the apoptosis of the cancer cells by DNA damage response [11], which is suppressed after silencing of the Keap1 [12], and promotes autophagy at a certain extent [13]. Everolimus is an mTOR inhibitor, disrupt tumor proliferation under overexpression of RB1CC1, which is a recently identified tumor suppressor implicated in autophagic and apoptosis [14]. Sunitinib, Cabozantinib, and Pazopanib are kinase inhibitors also employed in the treatment of RCC, each characterized by distinct mechanisms of action. Sunitinib, functioning as a multitargeted tyrosine kinase inhibitor, impedes tumor cell growth and angiogenesis by targeting receptors such as platelet-derived growth factor receptor (PDGFR), vascular endothelial growth factor receptor (VEGFR), and KIT receptor tyrosine kinase (KIT) [15]. Cabozantinib, as a multitargeted receptor tyrosine kinase (RTK) inhibitor, acts upon Mesenchymal Epithelial Transition Factor (MET), VEGFR, and AXL receptor tyrosine kinase (AXL), thereby hindering tumor cell growth and angiogenesis while concurrently modulating the tumor microenvironment [16]. Pazopanib, also classified as a multitargeted RTK inhibitor, primarily affects receptors including VEGFR, PDGFR, and KIT. Its disruption of the VEGF pathway interferes with tumor cell blood supply and growth. These diverse mechanisms offer a range of options for the treatment of RCC, necessitating tailored therapeutic approaches based on individual patient profiles. It is imperative to note that these inhibitors may manifest distinct side effects during treatment, underscoring the importance of physicians’ consideration of individual patient characteristics in optimizing treatment strategies [17]. Accumulating evidence has indicated that PCD is strongly associated with resistance to target therapy because cancer cell survival and target therapy pathways have complex crosstalk [7]. Here, we briefly introduce the classification of cell death, and discuss the role of cell death-linked molecules in the progress, prognosis, and therapy of ccRCC, in order to provide directions for further research and treatment.

## Ferroptosis

Ferroptosis constitutes an iron-mediated PCD, in which Lipid peroxidation coupled with free iron accumulation comprises the two key signals of membrane oxidative damage [18].

### Lipid peroxidation

Unrestricted lipid peroxidation mediates cell death in ferroptosis. Acyl-CoA Synthetase long-chain family member 4 (ACSL4) along with Lysophosphatidylcholine acyltransferase 3 (LPCAT3) are two regulatory factors in the biosynthesis of PUFA-PLs (polyunsaturated fatty acid-containing phospholipids) [18]. When ACSL4 expression was inhibited, PUFA-PLs were rapidly transformed into an acyl group of short-chain monounsaturated fat, making cells desensitized to feroptosis [19]. Certain lipoxygenases (LOXs) constitute a kind of non-heme iron-dependent enzyme, which can catalyze the oxidation of polyunsaturated fatty acids (PUFAs) to malondialdehyde and 4-hydroxynonenal, thus promoting the occurrence of ferroptosis [20].

### Antioxidant defense

The canonical Glutathionperoxidase-4 (GPX4) regulated ferroptosis pathway plays a vital regulatory role in scavenging intracellular ROS [18]. GPX4 has the ability to directly decrease lipid hydroperoxides, acting as a crucial suppressor of ferroptosis [21]. Repression of the cystine/glutamate transporter (system Xc^-^) or glutathione (GSH) can also lead to ferroptosis [22].

### Iron toxicity

In the presence of ROS and other oxidizing substances, Fe^2+^ can accelerate lipid oxidation in the cell membrane through Fenton reaction [23]. Factors conducive to the increase of intracellular Fe2+ content, for instance transferrin receptor (TfR) triggered iron transport, as well as autophagy released Fe^2+^ can enhance iron death [18].

### Ferroptosis and ccRCC

**Fig. 1:**
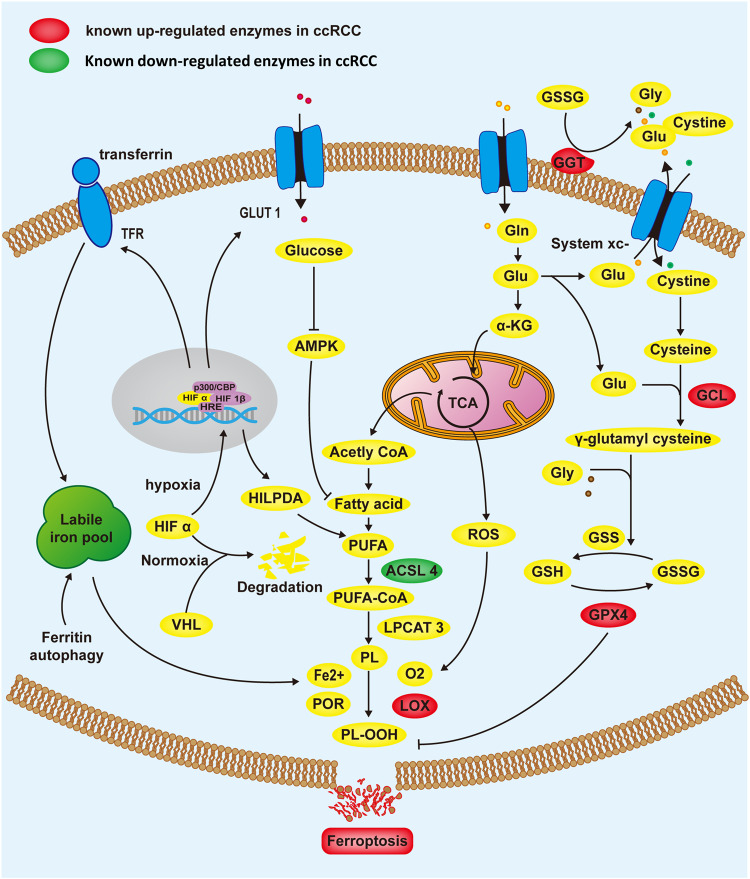
Metabolism and cell signaling associated with ferroptosis in ccRCC cell. The figure illustrates the relationship between HIF, ferroptosis, and metabolic pathways in ccRCC. HIF is involved in the metabolic reprogramming of ccRCC cells under hypoxia, and one of the “side effects” is that it increases tumor cells’ vulnerability to ferroptosis through a variety of metabolism pathways. To resist ferroptosis, ccRCC cells stimulate glutathione (GSH) production by raising the expression of glutathione metabolism target genes and inhibit acyl-CoA synthetase long chain family member 4 (ACSL4) expression. Abbreviations: TfR transferrin receptor, ROS reactive oxygen species, TCA mitochondrial TCA cycle, Gln glutamine, Glu glutamate, α-KG α-ketoglutarate, Gly glycine, PUFA polyunsaturated fatty acids, GSSG oxidized glutathione, GPX4 glutathioneperoxidase-4, GLUT1 Glucose transporter 1, LPCAT3 lysophosphatidylcholine acyltransferase 3, POR cytochrome P450 oxidoreductase, LOX lipoxygenase, GGT γ -glutamyl transferase, GCL glutamate cysteine ligase, GSS GSH synthetase.

Ferroptosis is closely related to metabolism. Up to now, ferroptosis is mainly regulated by cysteine-glutathione redox axis, lipid metabolism, iron metabolism, and glucose metabolism (Fig. 1).

### Glutathione metabolism

Reprogramming of glutamine metabolism is a prominent feature of ccRCC [2]. Metabolomics profiling revealed that GSH metabolism-linked metabolites, consisting of cysteine along with GSH, are more prevalent in advanced ccRCC than in normal kidney, and are linked to poorer survival outcomes in ccRCC patients [24]. Direct dampening of GSH synthesis can lead to ferroptosis in ccRCC cells, while restoration of VHL expression reverts ccRCC cells to oxidative metabolism, and renders them unresponsive to ferroptosis [25].

The system Xc^-^ antiporter constitutes a transmembrane protein complex composed of the SLC7A11 and SLC3A2 subunits and is primarily responsible for cystine absorption in exchange for intracellular glutamate [26]. Cancer cells appear to regulate their redox state along with metabolism via system Xc^-^ to enter a peroxide state and thus promote tumor growth [22]. Bioinformatics for comprehensive analysis of ccRCC found that SLC7A11 overexpression is linked to overall dismal survival [27]. At present, scientists have observed that SLC7A11 induces different cell death patterns in different tumor cells. In pancreatic duct adenocarcinoma, deletion of the System ${{\rm{X}}_{\rm{c}}}^{-}$ subunit, SLC7A11, triggered tumor-selective ferroptosis [28]. While in VHL-deficient ccRCC, Tang et al. found that inhibitors of SLC7A11 (sulfasalazine) triggered mixed-lineage kinase domain-like protein (MLKL)-mediated necroptosis [29]. In this study, the depletion of cystine resulted in a significant reduction of glutathione (GSH) levels, reaching an undetectable state However, researchers didn’t further detect markers of ferroptosis in ccRCC cells. Considering the complex regulatory net-works between different cell death signaling pathways, whether inhibition of SLC7A11 subunit induces ferroptosis in ccRCC remains to be further studied.

The γ -glutamyl cycle involves both de novo biosynthesis and degradation of GSH, with biosynthesis primarily dependent on two enzymes, glutamate cysteine ligase (GCL) and GSH synthetase (GSS), and degradation primarily dependent on one enzyme, GGT (glutamyl transferase), located in the outer membrane [30]. Li et al. found that the elevated expression along with escalated enzymatic activity of GCL is remarkably linked to ccRCC [31]. Another study found that elevated serum GGT is a sensitive biologic signature of metastatic ccRCC and is linked to a reduced survival rate in ccRCC patients [32]. GPX4 is the major enzyme using GSH as the cofactor in catalyzing the reduction of PLOOHs in mammalian cells, hence serving as a central inhibitor of ferroptosis in cancer cells [21]. Lu et al. observed that KLF2 dampens cancer cell migration along with invasion via modulation of ferroptosis via GPX4 in ccRCC [33]. In vitro, GPX4 inhibitors ML210 and RSL3 effectively inhibit ccRCC cell proliferation [34]. Up to now, clinical research targeting the GSH/GPX4 pathway in ccRCC is still limited. We firmly believe that targeting the dysregulated pathways related to glutathione metabolism and oxidative stress represents a pivotal direction for future drug development in ccRCC. By developing therapeutic interventions that modulate these pathways, potential treatment strategies could focus on restoring redox balance, enhancing oxidative stress responses, and potentially sensitizing ccRCC cells to conventional therapies or immunotherapies. Additionally, exploring novel targeted therapies that specifically interfere with the altered glutathione-dependent mechanisms could hold promise in overcoming drug resistance and improving overall treatment outcomes for ccRCC patients. However, further research and preclinical studies are warranted to validate the efficacy and safety of such targeted approaches before their translation into clinical practice.

### Lipid metabolism

Untargeted lipidomic analyses revealed that PUFAs were significantly accumulated in ccRCC compared to normal tissue [35]. The buildup of PUFAs suggests that ccRCC may be susceptible to ferroptosis, indicating that the membrane modification enzyme expression cannot be excessive. Subsequent studies proved that ACSL4 was down-regulated in ccRCC cells, and LPCAT3 showed no differential expression in ccRCC [36, 37]. Moreover, the expression level of ALOX15, one of the key enzymes capable of directly oxygenating PUFAs and PUFA-containing lipids in cell membranes, decreased with increasing tumor malignancy but remained consistently higher than that of normal tissues [38]. Therefore, based on the findings and observations in our study, we put forth the speculation that ferroptosis, a form of iron-dependent cell death, and lipid metabolic pathways are intricately linked and play a significant role in ccRCC pathogenesis. This association arises from the crucial role of lipid peroxidation, which is a hallmark of ferroptosis, in ccRCC tumorigenesis and progression.

### Hypoxia signaling

Hypoxia-inducible factors (HIFs) is an important inducer in the carcinogenesis of ccRCC, which leads to the expression of downstream genes in adaptation to hypoxic environment [5]. Ferroptosis is suppressed by glucose deprivation by activating AMPK, whereas HIF1α enhances glucose absorption in ccRCC cells to meet cell proliferation, while simultaneously increasing cell vulnerability to ferroptosis in the process [39, 40]. According to a recent study, ccRCC were highly sensitive to ferroptosis caused by GPX4 inhibition, and this sensitivity was driven by HIF 2α through PUFA lipid remodeling via hypoxia-inducible lipid droplet-associated protein [34]. This result suggests that HIF 2α may inhibit hypoxic tumor growth. In addition, the expression of HIF-1α under hypoxic conditions stimulates TfR, which means that iron content in ccRCC cells will increase [41]. These features imply that individuals with ccRCC may benefit from iron-chelating medications or agents that promote iron-mediated toxicity. It is worth noting that the United States Food and Drug Administration has approved belzutifan (a HIF-2α inhibitors) for the treatment of advanced ccRCC patients who have experienced disease progression after receiving PD-1/PD-L1 inhibitors and vascular endothelial growth factor tyrosine kinase inhibitors (VEGF-TKIs) [42].

### Ferroptosis related drugs in ccRCC

Depriving glutamine along with cysteine represents an opportunity for ccRCC VHL/HIF-linked therapy [7]. Erastin, as a system Xc^-^ repressor, has been shown to trigger ferroptosis in ccRCC [21]. Everolimus (mTOR repressor) is indicated as a second-line treatment for patients progressing on VEGF-inhibit therapy [43]. A recent research investigation documented that Everolimus can accelerate Erastin-triggered ferroptosis in ccRCC, demonstrating that Everolimus combined with Erastin is a possible treatment option for ccRCC treatment [44]. Moreover, the glutaminase inhibitor CB-839 was observed to enhance anti-tumor activity in combination with Cabozantinib or everolimus [45].

Sorafenib is a VEGFR-TKI inhibitor employed in treating refractory liver cancer and advanced ccRCC, but whether it is a ferroptosis inducer remains controversial. Dixon et al. found that sorafenib could dampen system Xc^-^, induce stress and cell ferroptosis [46]. Wang et al. argued that sorafenib did not trigger ferroptosis by dampening system Xc^-^ or some unidentified mechanism related to the GSH–GPX4 axis [47]. Recent study observed that other kinds of cell death can also lead to intense lipid peroxidation [48]. Therefore, drugs like sorafenib, which has the potential to affect multiple cell death signaling pathways, should be used with greater caution as a system Xc^-^ inhibitor.

Artemisinin, extracted from the plant Artemisia annua, has shown chemical sensitization in a variety of tumor cells. Zhu et al. revealed that artemisinin can induce ferroptosis by stimulating ROS production, elevating the intracellular free iron levels, and suppressing the antioxidant defense system [49]. Therefore, we believe that artemisinin may be an effective new drug for treating individuals with ccRCC.

## Immunogenic cell death

Immunogenic cell death (ICD) represents a distinct subset of PCD that possesses the unique ability to activate and stimulate the adaptive immune system against the antigens derived from dying cells. Actually, ICD activation depends on two main parameters: antigenicity and adjuvanticity [8]. Malignant cells can display antigenicity. Antigens such as tumor neoantigens and tumor-associated antigens are not perfectly covered by central tolerance [50]. Furthermore, damage-associated molecular patterns (DAMPs) usually act as adjuvants in ICD. Dying cells release a range of immunostimulatory DAMPs and cytokines to recruit antigen-presenting cells (APCs) and prime an adaptive immune response [51].

### DAMPs and cell death

**Fig. 2:**
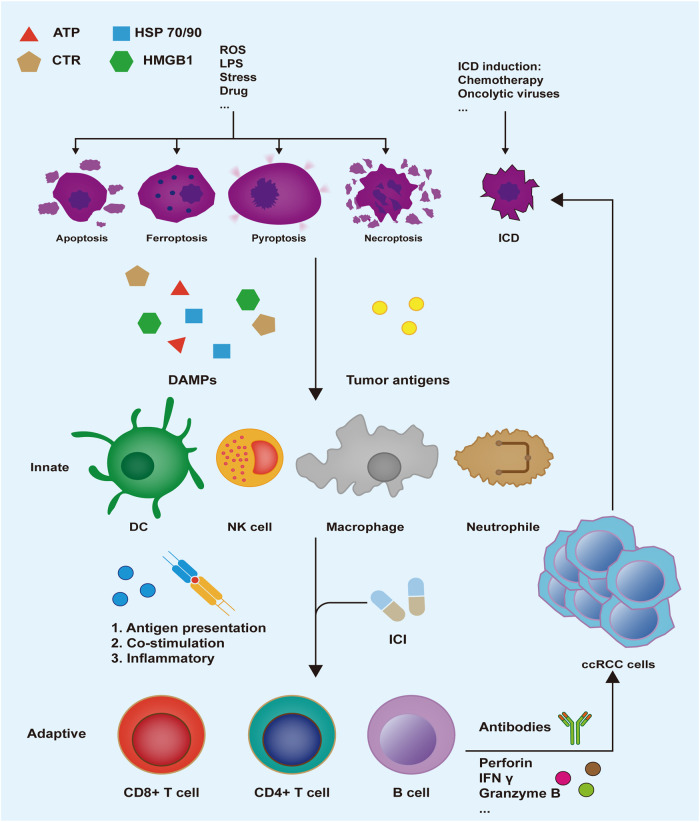
Heating up “cold” tumors. ccRCC cells that suffer immunogenic cell death (ICD) or other forms of cell death in response to drugs or other stresses can release tumor-associated antigens, cytokines, and damage-associated molecular patterns (DAMPs) that promote recruitment, phagocytic activity and maturation of innate immune cells. Among these cells, antigen-presenting cells (APCs) migrate to lymph nodes to prime adaptive immunity, therefore suppressing ccRCC tumor development and lowering the risk of metastasis. Abbreviations: LPS Lipopolysaccharide, CRT calreticulin, HMGB1 high-mobility group protein box 1, HSP heat shock protein, DC dendritic cell, NK natural killer cell, IFN interferon, ICI immune checkpoint inhibitor.

ICD offers a novel theoretical basis for improving the effectiveness of cancer therapy and relieving patients’ suffering. Except ICD inducers, ICD can be induced by other modes of cell death, which work as tumor vaccines, resulting in tumor‐distinct immune response (Fig. 2). Patients therefore benefit from cytotoxic chemotherapy and physically produce therapeutic responses over the long term.

The high-mobility group box 1 (HMGB1) protein can be released extracellularly in tumor cells with ferroptosis and act as a kind of DAMP to bind with receptors such as RAGE and TLR2, thus inducing immune cell activation coupled with the generation of cytokines [52].

The main substances released by pyroptosis are IL-1β, HMGB1, IL-18, along with ATP. IL-1β facilitates presentation of antigens between T lymphocytes and dendritic cells, promotes the differentiation of naive CD4+ T lymphocytes toward the Th17 phenotype [53]. IL -18 has the ability to stimulate immune cells to produce IFN-γ as well as to give NK cells an APC-like phenotype [54]. ATP is the main chemokine for macrophage migration, and a large amount of ATP released by pyroptosis is conducive to macrophage recruitment [55].

In 2016, Ages et al. first proposed that necroptosis is a kind of ICD [56]. Yatim et al. documented that RIPK1 expression, in conjunction with NF-κB activation during PCD, is essential for the initiation of adaptive immunity in CD8+ T cells [57]. CD8+ T cells are activated and effector cytokines are released by the immune response induced by necroptosis cells, which inhibits tumor proliferation in mice.

Apoptotic have long been considered to cause almost no immune response. Recent research, however, has shown linkages between some components of the immune system and apoptotic tumor cells, illustrating that a certain class of chemotherapeutic drugs, such as anthracyclines and mitoxantrone, might trigger an immunogenic kind of apoptosis [58]. Calreticulin is exposed to the plasma membrane as a DAME in early apoptosis and crosstalks with CD91 receptors in phagocytes, enabling them to ingest dead cells efficiently, resulting in subsequent cross-presentation of tumor antigen and tumor-distinct cytotoxic T lymphocyte responses [59].

### Immune checkpoint inhibitors and ccRCC

T lymphocytes are abundant in the ccRCC tumor. However, an excess of intratumoral T cells has been linked to high tumor grade and worse patient survival, indicating that the immune microenvironment is in an inhibitory status in ccRCC tumor [2].

Cytotoxic T lymphocyte protein 4 (CTLA4) exhibits elevated affinity for B7 antigens expressed on APC, therefore competes with the costimulatory receptor CD28 for B7 participation, thereby preventing the full activation of cytotoxic T cells [60]. Anti-CTLA4 has been utilized in combination immunotherapy with anti-PD1 for ccRCC. The combination of nivolumab plus ipilimumab showed longer duration of response and higher response rate in patients with advanced ccRCC [61].

Programmed cell death 1 (PD1) is another T cell activation inhibitor. Results of a phase III research trial in ccRCC patients showed that nivolumab increased overall survival to 25 months, in contrast with 19.6 months for mTOR inhibitor everolimus [62]. Recent trials have proposed a combination of anti-angiogenesis and targeted immunotherapy to overcome drug resistance. Pembrolizumab combined with axitinib demonstrated superior outcomes, including extended overall survival, progression-free survival, and a higher objective response rate when compared to sunitinib. Similarly, lenvatinib plus pembrolizumab exhibited significantly prolonged progression-free and overall survival. Additionally, the combination of nivolumab and cabozantinib showed substantial benefits over sunitinib, encompassing improvements in progression-free survival, overall survival, and the likelihood of treatment response [63–65]. The results show that this combination has a longer progression-free survival along with objective response rates.

### Immunogenic cell death inducer and ccRCC

Since the special ability of ICD to destroy cancer cells was established, ICD inducers have been a popular research topic. Chemotherapeutic drug-induced ICD can break through the drug resistance barrier, stimulate tumor-specific T cell immune response, and enhance the anti-tumor effect of immunotherapy, making it a novel approach for ccRCC [66]. Anthracycline is a classic ICD inducer. Mei et al. devised a liposome carrier for the co-delivery of a promising ICD stimulus (mitoxantrone) plus an inhibitor of the IDO-1 (indoximod) [67]. In ccRCC (RENCA) animal models, researchers observed a highly remarkable tumor volume reduction and an increase in calreticulin, perforin, and NKp46 staining [67]. In summary, a synergistic drug combination delivered by a custom-designed liposome is capable of turning non-inflamed or cold tumors hot, and the combination of ICI may further enhance the tumor killing effect.

Oncolytic viruses, as emerging ICD inducer, recruits and activates T cells by stimulating tumor cells to release DAMPs, thus breaking tumor immune tolerance, stimulating anti-tumor immunity, and killing cancer cells without harming normal cells [68]. Reovirus-mediated oncolysis and chemokine production were observed following ccRCC infection [69]. This gives researchers new therapeutic strategies. Keith et al. repurposed Sunitinib in combination with reovirus to further reduce the tumor burden, lessen immunosuppressive cell accumulation, and establish protective immunity against ccRCC [70].

## Apoptosis

**Fig. 3:**
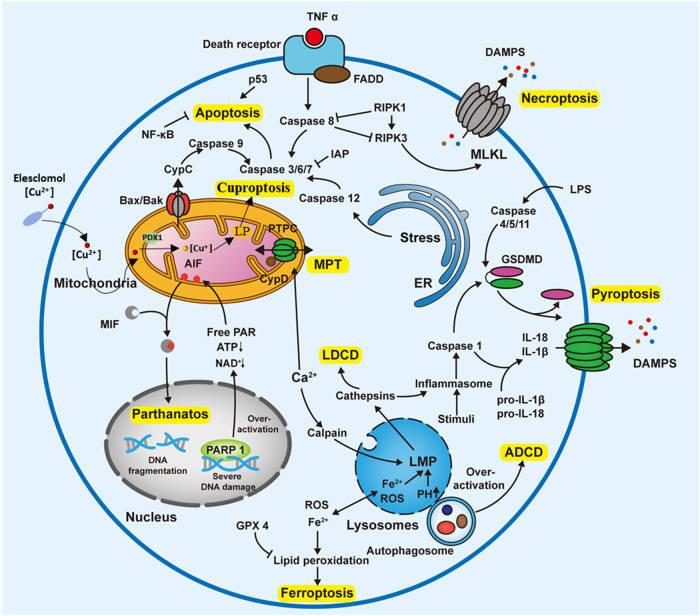
Some mechanisms of cell death in ccRCC cell. Abbreviations: ER endoplasmic reticulum, PARP 1 Poly (ADP-Ribose) polymerase 1, ADCD Autophagy-dependent cell death, LDCD Lysosomal-dependent cell death, LPS lipopolysaccharide, MLKL mixed-lineage kinase domain-like protein, LMP lysosomal membrane permeabilization, MPT mitochondrial permeability transition, GPX 4 Glutathionperoxidase-4, DAMPs (damage-associated molecular patterns), PARP1 (Poly (ADP-Ribose) polymerase 1), RIPK3 (receptor-interacting serine/threonine protein kinase 3), RIPK1 (receptor-interacting serine/threonine protein kinase 1), LP (lipoylated proteins).

Apoptosis is a cysteine protease induced death of cells that can be categorized into extrinsic apoptosis and intrinsic apoptosis (Fig. 3). Caspases are critical components of the apoptosis process as they are both the executors and effectors of apoptosis [8].

Extrinsic apoptotic pathway (death receptor dependence) is initiated by the interaction of exposed death receptors on the cell surface with their corresponding protein TNF family ligands [8]. When stimulated with the appropriate ligand, the receptor reveals its cytoplasmic death domine to recruit adapter proteins (FADD/TRADD) and pro-caspase-8 and/or -10, thus generating the death-inducing signaling complex, resulting in processing along with activation of downstream effector caspases, eventually inducing cell death [71].

Intrinsic apoptotic pathway (mitochondrial dependence) is mediated by intracellular signals in response to different conditions of stress [8]. Internal stimuli activate pro-apoptotic Bcl-2 family (Bak, Bax), resulting in the disruption of mitochondrial outer membrane permeability, allowing proteins, for instance cytochrome-C to diffuse into the cytoplasm, promoting the generation of apoptosome and activating pre-caspase-9, ultimately leading to apoptosis [71].

### Apoptosis and ccRCC

#### NF-κB

As a nuclear transcription factor, the sub-unit of NF-κB can generate an array of homo, as well as heterodimers. Activation of NF-κB drives the transcription of numerous anti-apoptotic genes of the Bcl-2 family consisting of Bcl-xl and Bcl-2 [72]. The way VHL regulates NF-κB activity remains unclear. Nevertheless, researchers discovered that pVHL-deficient ccRCC cells were resistant to TNF-α and bortezomib induced apoptosis, whereas exogenous introduction of the pVHL sensitized these cells to apoptosis by down-regulating NF-κB [72]. NF-κB also triggers multidrug resistance pathways. Sheng et al. found that silencing MUC13, a protein mediated by NF-κB, reverses ccRCC resistance to sorafenib and sunitinib [73].

#### P53

Downregulation of the protein cross-linking enzyme TGase2 stabilizes p53 in parallel with the induction of a 3–10-fold elevation in apoptosis for RCC cell lines [74]. Streptonigrin, a TGase2 inhibitor, has been shown to inhibit tumor proliferation via p53 mediated apoptosis of ccRCC [74]. Moreover, angiopoietin-like protein 3 can block the nuclear import of FAK, weaken p53 ubiquitination overexpression, promote ccRCC cell apoptosis and enhance sorafenib resopnse [75]. Mouse double minute-2 (MDM2), a protein-body that mediated degradation of p53 via ubiquitination, is an independent risk factor for ccRCC [76]. MDM2 antagonist Nutlin-3 elevates growth arrest, as well as p53 dependent senescence in ccRCC cells [77].

#### Bcl-2 family

The Bcl-2 family can be stratified into anti-apoptotic proteins along with pro-apoptotic proteins. Current studies mainly induce tumor apoptosis by inhibiting BCL family anti-apoptotic members [78]. ABT-737 along with ABT-263 are typically Bcl-2 homology 3 (BH3) mimetics that inhibit Bcl-xL, Bcl-2, as well as Bcl-w effectively while avoiding binding to Mcl-1 [79]. Mcl-1 expression causes ABT-737 resistance, and combination therapy is a plausible method for overcoming ABT-737 resistance in ccRCC. Aspirin and Cafestol have been shown to increase ABT-737 sensitivity to ccRCC cells by down-regulating Mcl-1 expression [80, 81]. In addition, TW-37, a second-generation BH3 mimetic, can downregulate Mcl-1 and synergically induce intrinsic apoptotic in ccRCC cells with the combination of ABT-263 [82].

## Pyroptosis

Pyroptosis is a Gasdermin-dependent form of PCD (Fig. 3) [8]. Activation pathways include classical and nonclassical pathways. In the classical pathway, intracellular pattern recognition receptors (PRRs) recognize drugs, DAMPs, and other signals, binding and activating the precursor of Caspase-1 through the adapter protein. Subsequently, GSDMD is cleaved by activated Caspase-1, and the n-terminal fragment of GSDMD aggregates and perforates the cell membrane, releasing a large number of inflammatory factors, resulting in pyroptosis [83, 84]. In the nonclassical pathway, caspase-4/5/11 activated by lipopolysaccharide (LPS) can cut GSDMD and trigger pyroptosis [85].

### Pyroptosis and ccRCC

GSDMD-mediated pyroptosis might influence the immune microenvironment of ccRCC, which means that GSDMD can be used as a potential therapeutic target [86]. The inflammasome serves as an activation platform for caspase-1during pyroptosis, and the bioinformatics analysis showed that NLRP3 expression in ccRCC was inversely linked to the degree of immune cell infiltration [87]. Tan et al. revealed that BRD4 inhibitors can dampen the proliferation along with epithelial-mesenchymal transition of ccRCC through NLRP3 inflammasome mediated pyroptosis [88]. Activation of GSDMs in normal tissues and immune cells may lead to severe tissue damage. To reduce the damage caused by pyroptosis to normal tissues, Wang et al. designed a targeted nanomaterial. The researchers combined the pre-cut GSDMA3 (NT + CT) with the cancer-imaging probe Phenylalanine trifluoroborate (Phe-BF3), and released GSDMA3 upon arrival at the specified location, resulting in the selective pyroptosis of tumor cells [89].

## Necroptosis

Necroptosis represents a kind of caspase-independent cell death triggered by receptor-interacting serine/threonine protein kinase 1 (RIPK1) (Fig. 3) [8]. In general, TNF-α binds to TNFR1 on cell membranes, attracting downstream protein molecules to form complex I [90]. Polyubiquitination RIPK1 in complex I can activate NF-κB and MAPK signaling pathways, promoting cell survival and inflammatory responses [90]. When ubiquitination of each component of complex I is repressed, it can be released from the cell membrane and form complexII, which activates caspase-8, as well as induces apoptosis [90]. Nonetheless, in the absence of caspase-8 activation, receptor-interacting serine/threonine protein kinase 3 (RIPK3) is mobilized to form the RIPK1-RIPK3 complex, which promotes MLKL formation and induces necroptosis [91].

### Necroptosis and ccRCC

The function of necroptosis in treating ccRCC is still unclear. RIPK1 along with RIPK3 expression were elevated in highly differentiated ccRCC cells [92]. When the NF-κB pathway was inhibited, ccRCC cells were more sensitive to IFN-γ-activated RIPK1-dependent necroptosis [93]. Wang et al. documented that emodin, a traditional Chinese herbal medicine, can induce necroptosis by activating the JNK signaling pathway [94].

## Parthanatos

Parthanatos is a Poly (ADP-Ribose) polymerase 1 (PARP1) dependent form of PCD. Oxidative stress-induced DNA damage activates PARP1 (Fig. 3) [8]. Hyperactivated PARP1 consumes a mass of ATP and NAD+ and binds to apoptosis-inducing factor (AIF), which mediates the transfer of AIF from the mitochondria to the nucleus, leads to partial chromosome lysis and cell death [95].

### PARP1 and ccRCC

PARP1, a Parthanatos-related component, contributes significantly to tumor proliferation, progression, and metastasis by assisting DNA repair pathways [96]. Researchers found that the development of ccRCC is linked to extreme aggregation of pADPr resulting from enhanced PARP1 expression and reduced PARG levels [96]. Therefore, PARP inhibitors are currently being explored in the management of ccRCC. Cellular experiments suggested that PARP repressors can significantly inhibit the proliferation of ccRCC cells [97]. Recent research evaluated the effects of 5F02, a non-NAD-like PARP1 inhibitor, and Olaparib on the viability of ccRCC cells and normal cells [98]. In comparison to the traditional PARP-1 inhibitor, 5F02 inhibited the proliferation of ccRCC cells with greater selectivity and effectiveness, while showing no significant toxicity to normal cells.

PBRM1 mutations are related to reduced immunological infiltrates and poor response to immune checkpoint blockade treatment, second only to VHL mutations in ccRCC [99]. Zhang et al. observed that PARP1 amplification was linked to increased immune cell infiltration along with the expression of immune checkpoint genes, indicating that patients with elevated PARP1 expression may benefit from ICI treatment [100]. However, the mechanism of PARP and ICI remains controversial owing to a dearth of high-quality studies. Hagiwara et al. reported that patients with PBRM1 mutation, the group with low PARP1 expression responded well to ICI treatment [101].

## Netotic cell death

NETotic cell death is the inflammatory cell death of neutrophils. Activated neutrophils are capable of trapping and killing pathogens via the release of extracellular NETs (Neutrophil extracellular traps) consisting of depolymerized chromatin and intracellular granular proteins [8]. Normally, pathogens, IL-8, and other inducers stimulate the neutrophils, leading to a rapid increase in intracellular ROS levels, promoting the production of Peptidearginine Deaminase 4 (PAD4), Myeloperoxidase (MPO), Neutrophil Elastase (NE) and in activated neutrophils [102]. Subsequently, the nuclear and granular membranes dissolve, and a significant number of antibacterial proteins in the cytoplasm attach to the depolymerized chromatin, forming a network structure that is eventually discharged into the extracellular space [102].

### Neutrophils and ccRCC

In recent years, the mechanism of neutrophils affecting tumors has emerged as a novel scientific ‘hotspot’. The NE released by NETs also changes the energy metabolism of tumor cells and promotes tumor growth [103]. Serum NETs levels can predict tumor metastasis to some extent. Wen et al. found that NETs generated by intravascular neutrophils enhance the coagulation system and shield circulating tumor cells (CTCs) from attack in ccRCC [104]. Moreover, CTCs are strongly linked with the expression of MPO, PAD4, and other critical NET-produced molecules in the peripheral blood of patients with ccRCC [104].

A recent study showed that neutrophil infiltration in the tumor microenvironment enhances ccRCC metastasis, and is associated with primary angiogenesis, leading to the growth of primary and metastatic ccRCC [105]. Chemokine CXCL7 produced by neutrophils in ccRCC is a risk factor for tumor progression and has been proven to be an effective marker for first-line therapy of metastatic ccRCC with sunitinib in plasma [106]. Increased neutrophil count over the upper limit of the normal peripheral blood neutrophil count is one factor contributing to the deterioration in overall survival in patients with metastatic ccRCC treated with targeted therapies [107]. Similar outcomes were seen in patients with metastatic ccRCC treated with ICI, with a higher peripheral blood neutrophil-lymphocyte ratio and a worse prognosis [108].

## Cuproptosis

Copper has a broad and important role in biological systems, acting as a cofactor in multiple enzyme active sites, and it is involved in various physiological activities such as oxidative stress, lipid metabolism, and energy metabolism [109]. As with other trace elements, once the concentration of intracellular copper ions exceeds the threshold, copper becomes toxic, causing cell death, but the mechanism of copper-induced cytotoxicity remains currently unknown [110]. The new term “cuproptosis” have been firstly defined by Tsvetkov et al. in 2022. Their original research proved that the occurrence mechanism of copper toxicity varies from all other known cell death mechanisms, including apoptosis, ferroptosis, pyroptosis, and necroptosis, and referred to targeting lipoylated TCA cycle proteins as the crux of this cell death mechanism. Proteotoxic stress responses triggered by the aggregation of lipoylated proteins (Fig. 3) and destabilization of Fe-S cluster proteins are responsible for copper-mediated cell death [111]. Copper ionophiles represented by elesclomol, which binds to ferric reductase 1 (FDX1) to promote cuproptosis, have been widely investigated in cancer treatment since 2019 [112].

### Cuproptosis and ccRCC

Researches on the association of cuproptosis to ccRCC treatment is still in its infancy. Several models based on three large-scale databases and clinical queues indicate cuproptosis-related genes are vital in tumor immunity and hold great potential for predicting OS of ccRCC patients. The Cuproposis score was strongly correlated with the infiltration levels of various immune cells in the TME [113, 114]. As a critical regulator of cuproptosis, FDX1 sparked greater interest in favorable prognosis in ccRCC than other individual prognostic parameters [115, 116]. Similarly, cuproptosis-related lncRNA also show pretty predicting potential [117].

## Lysosomal-dependent cell death

Lysosomal-dependent cell death (LDCD) is a form of PCD mediated by lysosomal membrane permeabilization (LMP) releasing cathepsins into the cytosol (Fig. 3) [8]. ROS-mediated lysosomal instability can induce LDCD, because the lysosomes lack antioxidant enzymes, so ROS can easily cross and destroy lysosomal membranes [118]. Cancer cells constantly increase their metabolic rate by altering the number, location, and activity of lysosomes to satisfy their growth and proliferation requirements. However, the changes indicated above promote cancer growth while simultaneously causing LMP and increasing the instability of lysosomes in ccRCC, which give us the therapeutic window [119].

CQ (chloroquine) and HCQ (hydroxychloroquine), V-Type H^+^-ATPase, agents interfering with sphingolipid metabolism, and HSP70 antagonists are four typical inducers of LMP [120]. In vitro, lysosomal-disrupting agents targeting the V-ATPase and raising lysosomal pH can significantly inhibit the proliferation of VHL-inactivated ccRCC cells [121]. CQ and HCQ are autophagy inhibitors that not only block the fusion of autophagosomes with lysosomes but also induce LMP by raising lysosome pH [120, 122]. Autophagy inhibitors are a treatment option to combine with mTOR inhibitors since therapeutic activation of autophagy is one mechanism of mTOR inhibitor resistance. [120, 123]. A clinical trial of everolimus plus HCQ showed that HCQ is an effective and low toxic autophagy inhibitor in patients with previously treated ccRCC [123].

## Autophagy-dependent cell death

Autophagy-dependent cell death (ADCD) relies exclusively on the autophagic pathway components, which is an important distinction because autophagy can also be involved in other forms of cell death (Fig. 2) [8]. Due to its tumor-initiating and tumor-suppressing effects, autophagy has various roles in cancer cells. ccRCC cell lines inherently exhibit an elevated basal level of autophagy [124]. STF-62247 induces ADCD independently of HIF-1 in ccRCC cells, and its combination with radiation enhances cell killing under aerobic, hypoxia, or physiological conditions [125].

## Mpt-driven necrosis

Mitochondrial Permeability Transition (MPT) is a physiological process that involves the opening of a non-selective pore in the inner mitochondrial membrane called the mitochondrial permeability transition pore (mPTP). This phenomenon plays a critical role in various cellular processes, including cell death, cellular homeostasis, and protection against mitochondrial damage. MPT-driven necrosis is a form of PCD that is triggered by particular perturbations of the intracellular microenvironment, for instance severe oxidative stress and cytosolic Ca2+ overload (Fig. 2) [8]. MPT driven necrosis is closely associated with ischemia-reperfusion injury in normal tissue [126]. However, the same mechanism can’t be observed in solid tumor even though solid tumor typically generates areas of intratumoral hypoxia [126, 127]. One explanation is that hypoxic tumors inhibit MPT driven necrosis by limiting Pi and Ca2+ accumulation through Warburg effect [127]. Considering metabolic reprogramming constitutes a prominent feature of ccRCC, the relationship between ccRCC and MPT driven necrosis deserves further study.

## Directions with Pcd in the future of ccRCC therapy

Regarding the systemic treatment of advanced ccRCC, several clinical trials have been completed (Table 1). The study indicates that, under monotherapy, patients with ccRCC exhibit a higher response rate to Belzutifan. Simultaneously, the combination therapy of Nivolumab and Ipilimumab has increased the overall response rate by 10%. A series of clinical trials demonstrate that the current optimal first-line treatment strategy continues to be the combination use of targeted drugs and ICI.

**Table 1:** The main trials about the different types of cell death.

Trial Identifier	Stage	Title/characteristics	Treatment	Comment	Cell death Type
NCT00326898	Phase 3	Clear Cell Renal Cell Carcinoma Stage I-III Renal Cell Cancer	sunitinib, sorafenib	No benefit to adjuvant sunitinib or sorafenib in ccRCC population	Ferroptosis
NCT00378703	Phase 2	Clear Cell Renal Cell Carcinoma Recurrent Renal Cell Carcinoma	sorafenib, temsirolimus	Not significantly improve median progression-free survival	
NCT00126503	Phase 1 Phase 2	Chromophobe Renal Cell Carcinoma Clear Cell Renal Cell Carcinoma	sorafenib tosylate	No benefit in ccRCC population	
NCT02974738	Phase 1	Clear Cell Renal Cell Carcinom	Belzutifan	a high rate of disease control and durable responses	
NCT02885649	Phase 1	Clear Cell Renal Cell Carcinoma Stage I Renal Cell Cancer	enzalutamide	Terminated	Apoptosis
NCT01391130	Phase 2	Metastatic Clear Cell Renal Cell Carcinoma	Sunitinib	Terminated	Neutrophils
NCT01158222	Phase 2	Clear Cell Renal Cell Carcinoma Stage IV Renal Cell Cancer	Sunitinib	No Results	
NCT02917772	Phase 2	Clear-cell Metastatic Renal Cell Carcinoma	Nivolumab Ipilimumab	An approximately 10% improvement in ORR	Immunogenic cell death
NCT01408004	Phase 2	Clear Cell Renal Carcinoma	Everolimus Pazopanib	Did not result in prolonged progression-free-survival, fewer toxic effects, or improved quality of life	Immunogenic cell death
NCT03015740	Phase 1 Phase 2	Clear Cell Renal Cell Carcinoma Metastatic Kidney Carcinoma	Sitravatinib nivolumab	Demonstrated high clinical activity, manageable toxicity, and promising correlative immune effects	Ferroptosis Immunogenic cell death

Lipid metabolism and glutamine metabolism are both a prerequisite for ferroptosis and the main mechanism of ccRCC. Therefore, it is very likely that the action of ferroptosis on ccRCC operates through pathways related to the two metabolisms mentioned above. Classical ferroptosis inducer systemic inhibition (Erastin) and GPX 4 inhibition (RSL3) inhibit ccRCC cell proliferation by inhibiting GSH synthesis and promoting lipid peroxidation, respectively. In addition, the hippo pathway effector TAZ regulates ferroptosis by affecting the level of NOX4, and resulting lipid ROS in RCC [128]. Mechanistic researches centered around lipid metabolism and glutamine metabolism should become the mainstream. Accordingly, drugs, such as sorafenib, sulfasalazine, and artesunate, are being applied in clinical trials. Although the presence of drug toxicity and immune resistance, immunogenic cell death therapy still holds great applications in ccRCC. ICI-based therapy has become the first-line treatment option for ccRCC due to its highly immunogenicity. In order to improve the clinical efficacy of this therapy, the bulk multi-omics approach was to continuely develop for selecting the appropriate patients. ccRCC is prone to immune infiltration. Cell death, such as pyroptosis, can release DAMPS to alter the tumor immune microenvironment. A small number of tumor cells (<15%) undergoing pyroptosis was sufficient to activate the T-cell-mediated anti-tumor immune response and, as a result, eliminate the entire tumor transplanted into the mouse model [53, 89]. It may be worthwhile to explore the relationship between cell death and immune microenvironment of ccRCC, as well as the therapeutic benefit of ICI combination with agents targeting the cell death pathway. Tumor cells’ resistance to apoptosis is one of the predominant drug resistance factors in ccRCC. Silencing genes related NF-κB survival pathway can suppress ccRCC cell migration and invasion. Anti-apoptotic proteins from the Bcl-2 family can decrease drugs sensitivity to ccRCC cells. Consequently, future researches should be directed towards how to increase drug sensitivity through surmount apoptosis protection pathways. Cuproptosis is a recently systematically proposed copper-dependent regulatory mode of cell death, and its particular functions in ccRCC is unknown. The current studies is solely based on bioinformatics analysis and prediction. Therefore, the affection of cuproptosis-realsted pathways and molecules in ccRCC are future research hotspots. As for the other modes of cell death such as necroptosis, parthanatos, netotic cell death, LDCD, ADCD and mpt-driven necrosis, although they have related specific genes, they are still not the main direction of research. Further studies may concentrate on how these alterations in cell death signaling pathways contribute to the development of ccRCC and promote drug resistance. Another issue of concern is that exploration of cell death pathways often does not exist in isolation, and the connections and interactions between various pathways are still worth exploring. Therapies based on multicellular death pathways hold bright promise.

## Conclusion

A break through in the treatment of ccRCC was the transition from non-specific to strictly targeted therapy. At present, the prognosis of patients with advanced ccRCC has been greatly improved. However, the problems of tumor recurrence and drug resistance remain to be solved. PCD, as a current research hotspot, has been showing important results and plays an important role in development, tissue homeostasis, inflammation, immunity, and a variety of pathophysiological conditions. With deepening research in the field of PCD, it is believed that more death-related molecular markers and therapeutic targets will be discovered and used in clinics.
